# Src Homology 2 Domain-Containing Protein Tyrosine Phosphatase Promotes Inflammation and Accelerates Osteoarthritis by Activating β-Catenin

**DOI:** 10.3389/fcell.2021.646386

**Published:** 2021-04-09

**Authors:** Tenghui Tao, Danni Luo, Chenghao Gao, Hui Liu, Zehua Lei, Wenbin Liu, Chuankun Zhou, Dahu Qi, Zhenhan Deng, Xuying Sun, Jun Xiao

**Affiliations:** ^1^Department of Orthopedics, Tongji Hospital, Tongji Medical College, Huazhong University of Science and Technology, Wuhan, China; ^2^Department of Sports Medicine, The First Affiliated Hospital of Shenzhen University, Shenzhen Second People’s Hospital, Shenzhen, China

**Keywords:** SHP2, osteoarthritis, chondrocytes, β-catenin, MAPK, NF-κB

## Abstract

Osteoarthritis (OA) is a chronic articular disease characterized by cartilage degradation, subchondral bone remodeling and osteophyte formation. Src homology 2 domain-containing protein tyrosine phosphatase (SHP2) has not been fully investigated in the pathogenesis of OA. In this study, we found that SHP2 expression was significantly increased after interleukin-1β (IL-1β) treatment in primary mouse chondrocytes. Inhibition of SHP2 using siRNA reduced MMP3, MMP13 levels, but increased AGGRECAN, COL2A1, SOX9 expression *in vitro*. On the contrary, overexpression of SHP2 exerted the opposite results and promoted cartilage degradation. Mechanistically, SHP2 activated Wnt/β-catenin signaling possibly through directly binding to β-catenin. SHP2 also induced inflammation through activating Mitogen-activated protein kinase (MAPK) and nuclear factor κB (NF-κB) pathways. Our *in vivo* studies showed that SHP2 knockdown effectively delayed cartilage destruction and reduced osteophyte formation in the mouse model of OA induced by destabilization of the medial meniscus (DMM). Altogether, our study identifies that SHP2 is a novel and potential therapeutic target of OA.

## Introduction

Osteoarthritis (OA) is the most common chronic joint disorder characterized by increased pain and joint dysfunction. It was once regarded as a disease of purely mechanical cartilage degradation. However, increased research and knowledge have revealed it to be a complex condition affecting the whole joint (Glyn-Jones et al., 2015). The pathology of OA includes the loss of articular cartilage, hypertrophic differentiation of chondrocytes, subchondral bone remodeling, synovial inflammation, and osteophyte formation (Li et al., 2018). In such inflammatory condition, terminally differentiated hypertrophic chondrocytes abnormally release several kinds of growth factors, cathepsins, and matrix metalloproteases (MMPs), which disturb the coordinated coupling between osteoclastogenesis and matrix remodeling at the chondro-osseous front, leading to cartilage degradation (Naski et al., 1998; Uusitalo et al., 2000; Stickens et al., 2004; Nakashima et al., 2011). The most well-known mechanisms of OA include the increased expression of matrix-degrading enzymes like MMPs, a disintegrin and metalloproteinases (ADAMTS), and the degradation of COL2A1 (collagen, type II, alpha 1) and AGGRECAN (Hollander et al., 1995; Lark et al., 1995; Neuhold et al., 2001; Glasson et al., 2005). Recently, an increasing amount of new molecules have been reported to be responsible for the degeneration of articular cartilage, such as tyrosine kinase Fyn, complement C5, Yes-associated protein, and hypoxia-inducible factor-2α. Dysregulation of these proteins is correlated with abnormal signaling pathways, such as the Wnt, Mitogen-activated protein kinase (MAPK), and nuclear factor κB (NF-κB) pathway (Wang et al., 2011; Deng et al., 2018; Li et al., 2018). However, the exact molecular mechanisms crucial for OA remain to be fully elucidated, because there is still a lack of effective targets for the treatment of such disease. Exploring new pathogenic factors and accurate signaling pathways will pave the way and provide new strategies for developing novel medications for the disease.

SHP2, encoded by *Ptpn11*, is a ubiquitously expressed Src homology 2 domain-containing protein tyrosine phosphatase, which has been proved to be involved in several kinds of bone diseases (Kamiya et al., 2014). Mesenchymal stem cell (MSC)-specific SHP2 knock-out mice develop a distinct increased number of terminally differentiated chondrocytes, accompanied by severe chondrodysplasia and completely blocked osteogenesis, resulting in various skeletal defects including postnatal growth retardation, delayed closure of the skull, pectus carinatum, and limb deformity. Consistently, the MAPK and protein kinase B (Akt) signal pathways are also suppressed (Lapinski et al., 2013). Conditional knockout of SHP2 in osteoclasts significantly suppresses receptor activator of nuclear factor (NF)-κB ligand (RANKL)-mediated signaling and impairs osteoclast (OCL) precursor cells differentiation or maturation (Zhou et al., 2015). These studies strongly indicate the vital role of SHP2 in bone remodeling. Besides, several studies have been conducted about SHP2 in OA. For example, one study revealed that long non-coding RNA named MM2P can prevent SHP2-mediated dephosphorylation of STAT3, the activation of which promotes transcription of chondrocyte-specific protein *Sox9* and contributes to cartilage repairment (Bai et al., 2020). Recently, Keisuke reported that the *Ptpn11* gene is up-regulated in fibroblast-like synoviocytes of rheumatoid arthritis (RA) compared to that of OA (Maeshima et al., 2016). However, the effect of SHP2 on OA progress remains elusive.

In our study, we found SHP2 is accumulated in the primary chondrocytes after IL-1β stimulation. We further investigated the role of SHP2 in OA by regulating SHP2 expression both *in vitro* and *in vivo*. The results demonstrated that inhibition of SHP2 alleviated the cartilage destruction in chondrocytes by targeting β-catenin signaling. Consistently, SHP2 knockdown delayed cartilage degradation in OA animal model. This study provided a potential novel therapeutic target for treating OA.

## Materials and Methods

### Reagents and Antibodies

Recombinant mouse IL-1β was purchased from R&D Systems (Minneapolis, MN, United States). Lipofectamine 3000 reagent was obtained from Invitrogen (Waltham, MA, United States). Anti-SHP2, β-catenin, p38, p-p38, extracellular signal-regulated kinase (ERK), p-ERK, c-Jun N-terminal kinase (JNK), p-JNK, p65, p-p65, IκBα, p-IκBα, IκB kinase (IKK)-β and p-IKKα/β antibodies were supplied by Cell Signaling Technology (Beverly, MA, United States). MMP3, MMP13 and p-β-catenin(Y142) antibodies were obtained from Abcam (Cambridge, United Kingdom). p-β-catenin(S33/37/141), COL2A1, and AGGRECAN antibodies were purchased from Abclonal (Wuhan, China). GAPDH antibody was purchased from Boster (Wuhan, China) and secondary antibodies were obtained from the Jackson lab.

### Isolation and Culture of Primary Chondrocytes

Knee joint cartilages were dissected from newborn mice (5-days-old) to harvest the primary chondrocytes. After digestion with 0.2% trypsin for 30 min, cartilage pieces were purified and digested with 0.25% collagenase II dissolved in Dulbecco’s Modified Eagle Medium (DMEM)/F12 (Hyclone, United States) culture medium at 37°C for 6–8 h. The released chondrocytes were resuspended and cultured in DMEM/F12 supplemented with 10% fetal bovine serum (FBS) (Gibco, Waltham, United States), 100 units/ml penicillin and 100 μg/ml streptomycin at 37°C in an incubator. Cells were plated onto 10 cm plates and grown to approximately 80% confluences before each experiment.

### Small Interfering RNA and Plasmid Transfections

Transfections were performed using Lipofectamine 3000 reagent. Chondrocytes were plated onto six-well plates. For siRNA transfection, 50 pM siRNA (RiboBio, Guangzhou, China) or scrambled siRNAs, 5 μL of Lipofectamine 3000, 250 μL of Opti-MEM, and 2 mL of complete medium were mixed and added to each well. The siRNA (sequence 5′-GAACCTTCATTGTGATTGA-3′) targeting mouse SHP2 was purchased from RiboBio (Guangzhou, China). For SHP2 or active form of β-catenin (β-cat-S33Y) overexpression, chondrocytes were transfected with 1 μg SHP2 overexpression plasmids (OriGene, Rockville, MD, United States) or β-cat-S33Y in 2 mL complete medium, added with 5 μL Lipofectamine 3000 and 250 μL Opti-MEM. 48 h after transfection, chondrocytes were harvested and subjected to different experiments. The efficiency of knockdown or overexpression was confirmed by RT-qPCR and western blot.

### Cell Proliferation Analysis

Primary chondrocytes were plated onto 96-well plates for SHP2 siRNA or over-expressed plasmid transfection and then were stimulated with IL-1β for 24 h. All tests were performed in triplicate and cell proliferation was measured by Cell Counting Kit-8 Assay (CCK-8, TargetMol). To measure the proliferation rate, cells were cultured in the CCK-8 culture medium for 1 h, and then were subjected to absorbance measurement at 450 nm using a microplate reader (Bio Tek, United States).

### Flow Cytometry

An Annexin V-FITC Apoptosis Detection Kit (Keygentec, China) was applied to determine the apoptosis rates following the manufacturer’s instructions. Briefly, Collected chondrocytes were suspended in binding buffer in a concentration of 1 × 10^5^ cells/ml and were stained with 5 μl Annexin V-FITC and 10 μl PI for 15 min in the darkroom. The results were analyzed using a flow cytometer (Becton Dickinson, United States).

### Cell Migration Assay

A two-well Ibidi silicone culture insert (Ibidi, Martinsried, Germany) was set onto the middle of the well in 12-well plates, and 70 μL chondrocyte suspension with 5 × 10^5^ cells were seeded onto each well of the chamber. After cell attachment, the silicone insert was carefully removed, leaving a 500-μm cell-free gap. The chondrocytes were incubated for 24 h in the presence or absence of IL-1β, and images were captured at different time points using Nikon inverted microscope (Nikon, Tokyo, Japan).

### Animal Model

Animals were purchased from the Experimental Animal Center of Tongji Hospital, Huazhong University of Science and Technology. All the experiments were approved by the Ethics Committee on Animal Experimentation of the hospital. 12-weeks-old male C57BL/6J mice were used to induce OA by the destabilized medial meniscus (DMM) surgery which was performed as previously described (Glasson et al., 2007). To conduct animal experiments, a total of forty-eight mice were randomly divided into four groups: (1) Sham group: sham-operated mice administered with Ad-shControl adenoviruses (*n* = 6 for both 8 and 12 weeks); (2) Sham + Ad-shSHP2 group: sham-operated mice treated with Ad-shSHP2 adenoviruses (*n* = 6 for both 8 and 12 weeks); (3) DMM + Ad-shControl group: DMM-operated mice administered with Ad-shControl adenoviruses (*n* = 6 for both 8 and 12 weeks); (4) DMM + Ad-shSHP2 group: DMM-operated mice administered with Ad-shSHP2 adenoviruses (*n* = 6 for both 8 and 12 weeks). Half of the mice were sacrificed 8 weeks after surgery, and the other half were sacrificed 12 weeks after surgery. To perform the surgery, the mice were anesthetized and the right knee was prepared before surgery. Then, the joint capsule was opened and the fat pad was removed to expose the medial menisco-tibial ligament under the microscope. The ligament was cut before suturing the joint capsule and skin wound. In sham-operated groups, only the joint capsule was opened, while other tissues remained intact. The knees were dissected at 8 or 12 weeks after surgery, respectively after sacrificing. To knock down SHP2 *in vivo*, shRNA was designed and mixed by Vigene Biosciences Inc. (Shandong, China). 10 μL Ad-shSHP2 or Ad-shControl adenoviruses (1 × 10^9^ plaque-forming units [PFUs]) were injected into the right knee joint cavity 1 week after DMM surgery using a 33G needle (Hamilton, Bonaduz, GR, Switzerland) once a week for 8 or 12 weeks after surgery.

### RNA Extraction and RT-qPCR

Total RNA was extracted from cultured chondrocytes using TRIzol reagent (Takara Bio, Japan) according to the manufacturer’s descriptions. 0.5–1 μg RNA was used to synthesize cDNA using Revert Aid First Strand cDNA synthesis Kit (Toyobo, Osaka, Japan). Templates were amplified using the SYBR Green Quantitative PCR Protocol (TaKaRa, Shiga, Japan) to determine mRNA levels. All primer sequences used are listed below: *Shp2*, 5′-AGAGGGAAGAGCAAATGTGTCA –3′ (sense), 5′-CTGTGTTTCCTTGTCCGACCT-3′ (antisense); *Axin2*, 5′-ATGAGTAGCGCCGTGTTAGTG-3′ (sense), 5′-GG GCATAGGTTTGGTGGACT-3′ (antisense); *Lef1*, 5′-GCCA CCGATGAGATGATCCC-3′ (sense), 5′-TTGATGTCGGCTA AGTCGCC-3′ (antisense); *Tcf4*, 5′-GATGGGACTCCCT ATGACCAC-3′ (sense), 5′-GAAAGGGTTCCTGGATTGCCC-3′ (antisense); *Aggrecan*, 5′-AGGTGTCGCTCCCCAACTAT-3′ (sense), 5′-CTTCACAGCGGTAGATCCCAG-3′ (antisense); *Col2a1*, 5′-CTTCACAGCGGTAGATCCCAG-3′ (sense), 5′-ACCAGGGGAACCACTCTCAC (antisense); *Mmp3*, 5′-ACTC CCTGGGACTCTACCAC-3′ (sense), 5′-GGTACCACGAGGA CATCAGG-3′ (antisense); *Mmp13*, 5′-TGATGGACCTTCT GGTCTTCTGG-3′ (sense), 5′-CATCCACATGGTTGGGAAGT TCT-3′ (antisense); *Gapdh*, 5′-CTCCCACTCTTCCACCTTCG-3′ (sense), 5′-TTGCTGTAGCCGTATTCATT-3′ (antisense); *Sox9*, 5′-CAGCCCCTTCAACCTTCCTC-3′ (sense), 5′-TGATGGTCAGCGTAGTCGTATT-3′ (antisense); *Col10a1*, 5′-T TCTGCTGCTAATGTTCTTGACC-3′ (sense), 5′-GGGATGAA GTATTGTGTCTTGGG-3′ (antisense). The relative mRNA levels of target genes were calculated using the 2^–ΔΔCq^ method.

### Western Blotting Analysis

Cultured chondrocytes were lysed by ice-cold RIPA lysis buffer (Boster, Wuhan) containing 1% protease and phosphatase inhibitor for 30 min. Then, the cell lysates were immediately configurated at 10,000 g at 4°C for 30 min. Equal amounts of protein were loaded on SDS-PAGE (10–15%) for electrophoresis, and then the proteins were transferred to polyvinylidene fluoride (PVDF) membranes. Each membrane was blocked with 5% non-fat milk in Tris-buffered saline with 0.1% Tween 20 buffer (TBST) for 1 h and then incubated with primary antibodies overnight at 4°C on a shaker. After incubation with horseradish peroxidase-conjugated secondary antibodies for 1 h, proteins were visualized by an enhanced chemiluminescence kit (Thermo Fisher Scientific, Waltham, MA, United States) in the ChemiDoc XRS System (Bio-Rad Laboratories, Hercules, CA, United States). Proteins were quantified using Image lab software and the targeted protein levels were normalized to GAPDH or relative total proteins.

### Co-immunoprecipitation

The interactions of SHP2 with β-catenin proteins were detected in primary chondrocytes. Cells were lysed in lysis buffer (20 mM Tris, pH 7.5, 150 mM NaCl, 1% Triton, and 10% glycerol) containing 1% protease and phosphatase inhibitor. Lysates were pre-cleared with the control IgG for 1 h to remove any proteins that bind non-specifically to immunoprecipitation components. Then, the primary antibodies or relative IgG, and the protein A/G beads were added to the lysates, and the mixture was incubated overnight at 4°C for immunoprecipitation. After being rinsed five times with lysis buffer, the immunoprecipitates were boiled in the loading buffer and then were subjected to western blotting.

### Micro-Computed Tomography

After euthanasia, the right knee joints of the mice were harvested and fixed in 4% formaldehyde for 48 h. High-resolution micro-computed tomography (μCT, Scanco Medical, Bassersdorf, Switzerland) was used to analyze the subchondral bone structures at 15 μm resolution, 70 kVP and 112 μA x-ray energy. The three-dimensional images were reconstructed according to the manufacturer’s instructions. The parameters of Bone volume per unit total volume (BV/TV), Trabecular number (Tb.N), trabecular separation (Tb.Sp) and trabecular thickness (Tb.Th) were evaluated using the μCT system software.

### Histopathological and Immunocytochemical Analysis

After scanning, the tissues were fixed in 4% paraformaldehyde for 2 days and decalcified in 10% EDTA (pH 7.4) for 4 weeks. Subsequently, the tissues were embedded in paraffin and sectioned continuously at 5 μm thickness for hematoxylin and eosin (HE), toluidine blue and Safranin O/Fast Green staining. The images were taken and scored for degree of articular cartilage damage according to the Osteoarthritis Research Association (OARSI) histopathology scoring system in a blind manner (Glasson et al., 2010). For DAB or immunofluorescence staining, sections were deparaffinized, hydrated, and blocked with BSA containing 0.1% Triton X-100 for 1 h at room temperature. After incubation with primary antibodies against MMP3 (1:100), MMP13 (1:100), SHP2 (1:100), COL2A1 (1:200), AGGRECAN (1:100) overnight at 4°C, the sections were incubated with HRP-conjugated secondary antibodies and counterstained with hematoxylin or corresponded fluorescence secondary antibodies. Finally, all the images were taken under a microscope.

### Immunofluorescence

The co-expression analysis of SHP2 and β-catenin in chondrocytes were performed by immunofluorescence staining. After being fixed in 4% paraformaldehyde and permeabilized in 0.2% Triton X-100, the chondrocytes were incubated with primary antibodies against β-catenin (1:100) and SHP2 (1:100) overnight at 4°C. The cells were subsequently incubated with anti-mouse and anti-rabbit secondary antibodies for 1 h. Finally, all images were captured using a fluorescence microscope.

### Statistical Analysis

All data were presented as means ± SEM. Statistical analyses were conducted using GraphPad Prism v. 5.0 (Graphpad Software Inc., San Diego, CA, United States). All experiments were independently repeated at least three times. One-way ANOVA followed by LSD’s *post hoc* tests were used to test the differences among groups. The student’s *t*-test was used to assess statistically significant differences in the data between groups. Values of P < 0.05 were considered statistically significant.

## Results

### SHP2 Expression Is Increased in Primary Chondrocytes After IL-1β Treatment and in Mouse OA Cartilage

**FIGURE 1:**
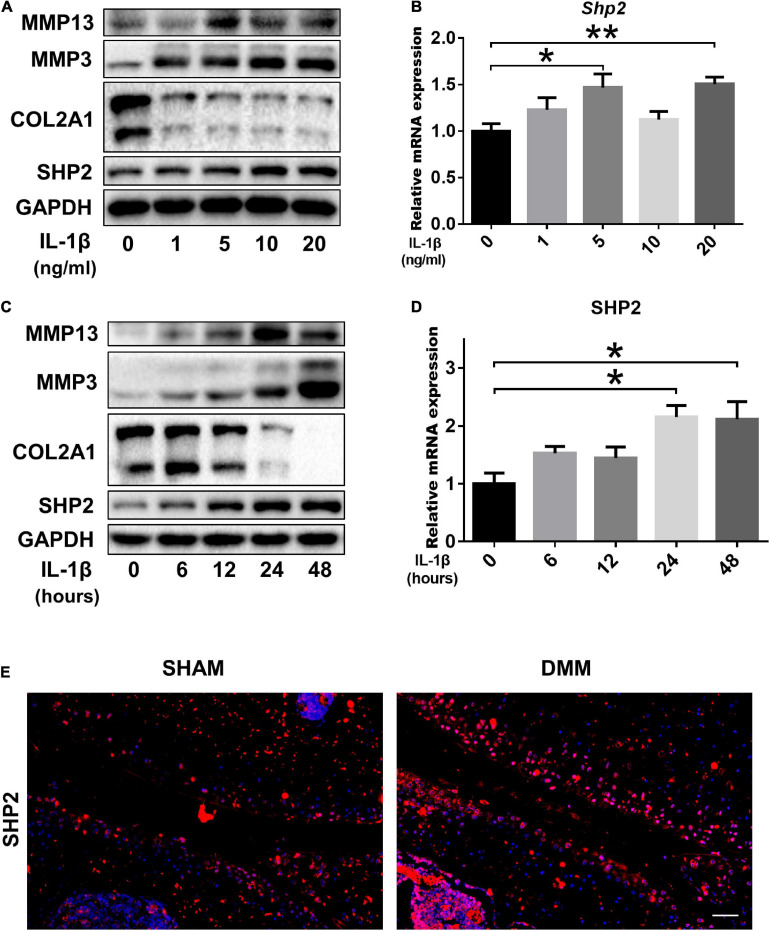
SHP2 expression is increased in primary chondrocytes after IL-1β treatment and in mouse OA cartilage. **(A)** Representative western blot of MMP3, MMP13, COL2A1, SHP2, and **(B)** mRNA level of *Shp2* determined by qRT-PCR in chondrocytes after stimulation with various doses of IL-1β for 24 h. **(C)** Representative western blot of MMP3, MMP13, COL2A1, SHP2, and **(D)** mRNA level of *Shp2* determined by qRT-PCR in chondrocytes after stimulation with 5 ng/ml IL-1β for different time points. *P < 0.05, **P < 0.01 versus chondrocytes without IL-1β treatment. **(E)** Representative immunofluorescence images of SHP2 in articular cartilage in DMM and Sham mice, scale bar = 100 μm.

To investigate the role of SHP2 in OA, cultured chondrocytes were exposed to different concentrations of IL-1β (0, 1, 5, 10, 20 ng/ml) to mimic OA model *in vitro*. Matrix-degrading enzyme MMP13 and MMP3 were gradually increased, while Col2a1 level was decreased upon IL-1β treatment in a dose-dependent manner, indicating a successful induction of inflammation in chondrocytes. Subsequently, SHP2 was elevated gradually by IL-1β (Figure 1A). Besides, the mRNA levels of *Shp2* were also increased after 5 and 20 ng/ml IL-1β treatment for 24 h although no obvious significance was observed at 1 and 10 ng/ml after IL-1β treatment (Figure 1B). We thus treated chondrocytes using 5 ng/ml IL-1β for different time points. Results showed MMP13 level increased within 24 h exposure but was slightly decreased at 48 h, SHP2 expression showed the same trend, MMP3 level elevated while COL2A1 decreased time-dependently within 48 h of treatment (Figure 1C). The mRNA level of *Shp2* was up-regulated in a time-dependently manner but was statistically significant at 24 and 48 h after IL-1β treatment (Figure 1D). Furthermore, we assessed the expression of SHP2 in the articular cartilage of DMM and Sham-operated mice by immunofluorescence staining, and the result revealed that SHP2 was strongly elevated in OA cartilage compared with the sham-operated mice (Figure 1E). Our data demonstrated that SHP2 was up-regulated both *in vivo* and *in vitro* in OA pathogenesis.

### SHP2 Regulates IL-1β–Induced Chondrocyte Destruction *in vitro*

**FIGURE 2:**
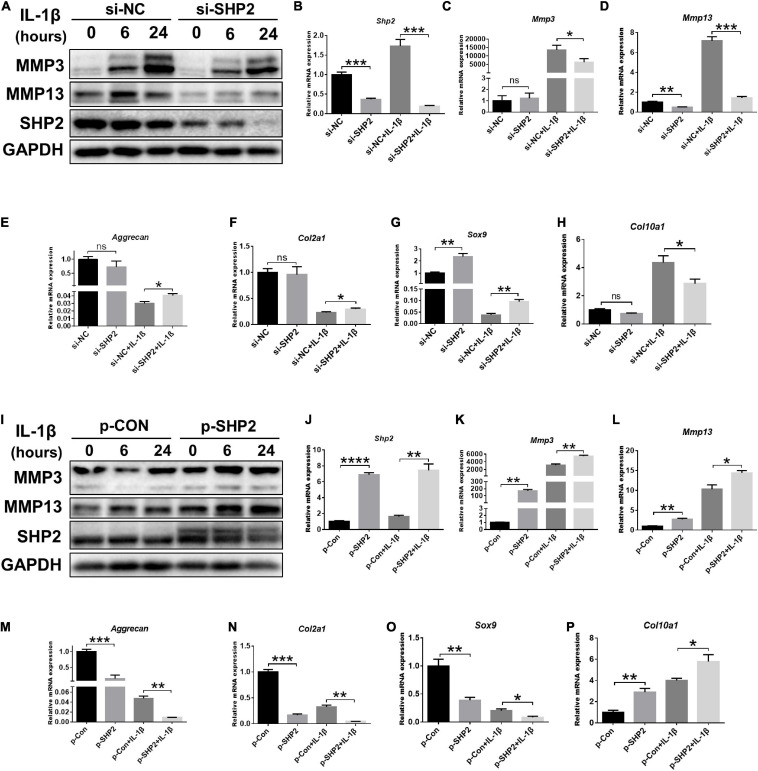
SHP2 regulates IL-1β–induced cartilage destruction *in vitro*. **(A)** Representative western blot of MMP3, MMP13 and SHP2 after chondrocytes were transfected with si-NC or si-SHP2 for 48 h and then were treated with IL-1β (5 ng/ml) for indicated time points. **(B–H)** mRNA levels of *Shp2*, *Mmp3*, *Mmp13*, *Aggrecan*, *Col2a*, *Sox9*, and *Col10a1* were shown in chondrocytes that were transfected with si-NC or si-SHP2 for 48 h and then were treated with IL-1β (5 ng/mL) for 24 h. *P < 0.05, **P < 0.01, ***P < 0.001 versus si-NC transfected chondrocytes. **(I)** Representative western blot of MMP3, MMP13 and SHP2 after chondrocytes transfected with control plasmid (p-CON) or SHP2 (p-SHP2) overexpressed plasmid for 48 h and then incubated with or without IL-1β (5 ng/ml) for indicated time points. **(J–P)** mRNA levels of *Shp2*, *Mmp3*, *Mmp13*, *Aggrecan*, *Col2a1*, *Sox9*, and *Col10a1* were shown in chondrocytes transfected with p-CON or p-SHP2 plasmids for 48 h and then stimulated with IL-1β (5 ng/mL) for 24 h. *P < 0.05, **P < 0.01, ***P < 0.001, ****P < 0.0001 versus p-CON transfected chondrocytes.

To further investigate the effect of SHP2 on IL-1β–induced chondrocyte destruction, SHP2 was knocked down using siRNA or overexpressed using plasmid in mice chondrocytes. The efficiency of SHP2 knockdown and overexpression was verified by qPCR and western blotting (Figures 2A,B,I,J). Subsequently, we performed CCK8 assays, flow cytometry and cell migration assays to test cell function. Results showed chondrocytes stimulated with IL-1β (5 ng/ml) for 24 h markedly increased the apoptotic rates which was not significantly influenced by SHP2 knockdown or overexpression (Supplementary Figures 1A–D). Furthermore, SHP2 knockdown or overexpression has no effect on chondrocyte viability (Supplementary Figures 1E,F). Cell migration assays showed scratch distances were not altered in SHP2 knockdown or overexpressed chondrocytes (Supplementary Figures 1G–J). These results suggested SHP2 had no significant effect on chondrocyte apoptosis, proliferation or migration. We then evaluated the role of SHP2 in IL-1β–induced chondrocyte degeneration. Downregulation of SHP2 decreased the elevation of MMP3 and MMP13 induced by IL-1β in both protein and mRNA levels (Figures 2A,C,D). *Aggrecan*, *Col2a1*, *Sox9*, and *Col10a1* levels were also tested by qPCR. The results showed that SHP2 knockdown increased *Aggrecan*, *Col2a1* and *Sox9* mRNA level, but decreased *Col10a1* mRNA level after IL-1β treatment in chondrocytes (Figures 2E–H). Furthermore, SHP2 overexpression distinctly increased the elevation of levels of MMP3 and MMP13 induced by IL-1β in protein and mRNA (Figures 2I,K,L). Consistently, *Aggrecan*, *Col2a1*, *Sox9* mRNA levels were reduced and *Col10a1* mRNA level was increased in chondrocytes exposed to IL-1β (Figures 2M–P). In short, our data displayed that SHP2 aggravated IL-1β–induced chondrocyte destruction *in vitro.*

### SHP2 Interacts With β-Catenin in Chondrocytes and Activates Wnt/β-Catenin Pathway

**FIGURE 3:**
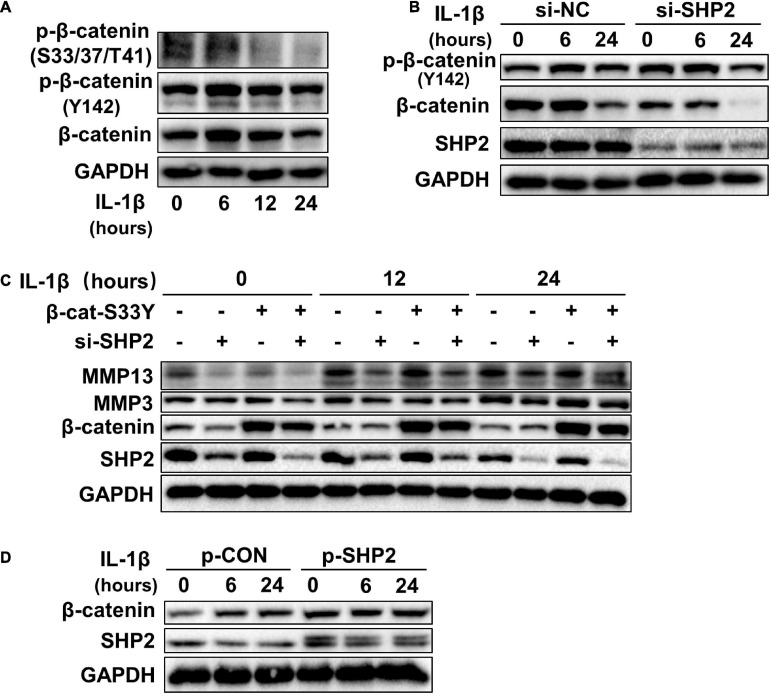
SHP2 activates Wnt/β-catenin in chondrocytes. **(A)** Representative western blot of p-β-catenin (S33/37/T41), p-β-catenin (Y142) and β-catenin, in chondrocytes incubated with IL-1β of 5 ng/ml for the indicated time points. **(B)** Representative western blot of p-β-catenin (Y142), β-catenin and SHP2 in chondrocytes incubated with IL-1β (5 ng/ml) for indicated time points after si-NC or si-SHP2 transfection for 48 h. **(C)** Representative western blot of MMP3, MMP13, β-catenin, and SHP2 in chondrocytes incubated with IL-1β (5 ng/ml) for indicated time points after siRNA or active form of β-catenin (β-cat-S33Y) transfection with for 48 h. **(D)** Representative western blot of β-catenin and SHP2 in chondrocytes incubated with IL-1β (5 ng/ml) for the indicated time points after p-CON or p-SHP2 transfection for 48 h.

**FIGURE 4:**
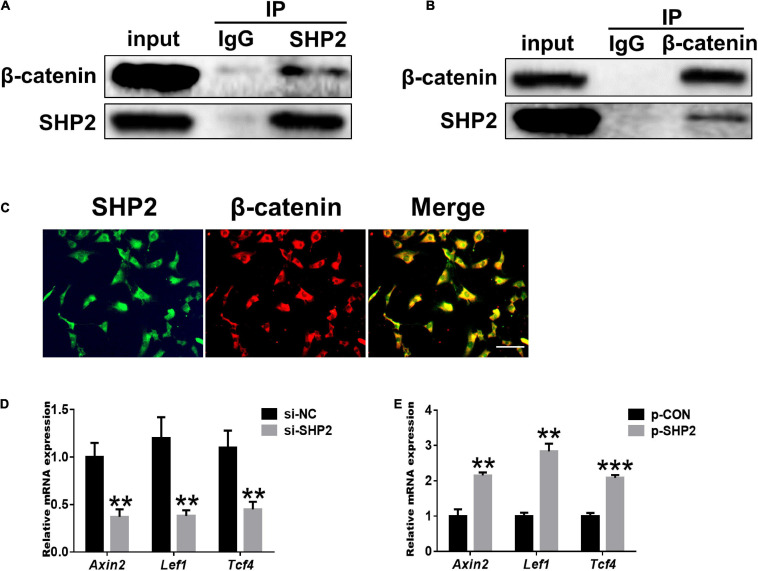
SHP2 interacts with β-catenin and activates Wnt/β-catenin target genes. **(A)** β-catenin was co-immunoprecipitated with an anti-SHP2 antibody and **(B)** SHP2 was co-immunoprecipitated with an anti-β-catenin antibody in chondrocytes. **(C)** Immunofluorescence image for SHP2 and β-catenin cellular localization in chondrocytes. Scale bar, 100 μm. **(D)** Relative mRNA levels of three Wnt/β-catenin targets *Axin2*, *Lef1*, and *Tcf4* in chondrocytes after si-NC or si-SHP2 transfection. **(E)** Relative mRNA levels of three Wnt/β-catenin targets *Axin2*, *Lef1*, and *Tcf4* in chondrocytes after p-CON or p-SHP2 transfection. *n* = 3, **P < 0.01, ***P < 0.001 versus si-NC or p-CON transfected chondrocytes.

Accumulating data identified that canonical Wnt/β-catenin signaling plays an important role in modulating the pathogenesis of OA (Wu et al., 2010; Bouaziz et al., 2015; Zhou et al., 2017). Activation of β-catenin resulted in the development of an OA-like phenotype (Zhu et al., 2009), while inhibition of β-catenin signaling in articular chondrocytes contributes to cartilage destruction (Zhu et al., 2008). Other studies found that SHP2 and β-catenin proteins can form a complex and SHP2 increased β-catenin accumulation by inhibiting glycogen synthase kinase 3β (GSK-3β)–mediated β-catenin degradation in liver cancer (Biswas et al., 2006; Xiang et al., 2017). To unveil the possible mechanism by which SHP2 promotes OA progression, we treated primary chondrocytes with IL-1β and found that IL-1β suppressed phosphorylated β-catenin at S33/37/141 sites, induced β-catenin phosphorylation at Y142, and increased total β-catenin expression at 6 and 12 h after IL-1β treatment. Although the expression of total β-catenin was slightly decreased 24 h after IL-1β treatment (Figure 3A). Downregulation of SHP2 by siRNA remarkably reduced β-catenin expression at 6 and 24 h after IL-1β treatment. However, the phosphorylated site at Y142 of β-catenin was not significantly changed in SHP2-downregulated cells after IL-1β treatment, indicating that SHP2 did not specifically dephosphorylate this site (Figure 3B). On the contrary, SHP2 overexpression promoted the expression of β-catenin (Figure 3D). To confirm SHP2 induced overexpression of matrix-degrading enzymes was indeed through β-catenin, we transfected mouse chondrocytes with plasmids expressing an active form of β-catenin with a mutation on the S33 site (β-cat-S33Y) (Ming et al., 2012). Overexpression of β-cat-S33Y prominently reversed the downregulation of MMP13 and MMP3 caused by SHP2 knockdown at 24 h after IL-1β treatment (Figure 3C). Additionally, the interaction of SHP2 and β-catenin in the primary chondrocytes was identified by co-immunoprecipitation (CO-IP) (Figures 4A,B). Besides, double staining for β-catenin and SHP2 was also performed and SHP2 colocalized with β-catenin in chondrocytes (Figure 4C). We further examined the target genes of the canonical Wnt/β-catenin pathway such as *Axin2*, *Lef1*, and *Tcf4*. In accordance with the β-catenin expression, the mRNA levels of these genes were attenuated by knocking down SHP2, but were increased significantly after SHP2 overexpression in chondrocytes (Figures 4D,E). Together, our results suggested that SHP2 activated Wnt/β-catenin signaling in chondrocytes.

### SHP2 Promotes MAPK and NF-κB Signaling Pathways

**FIGURE 5:**
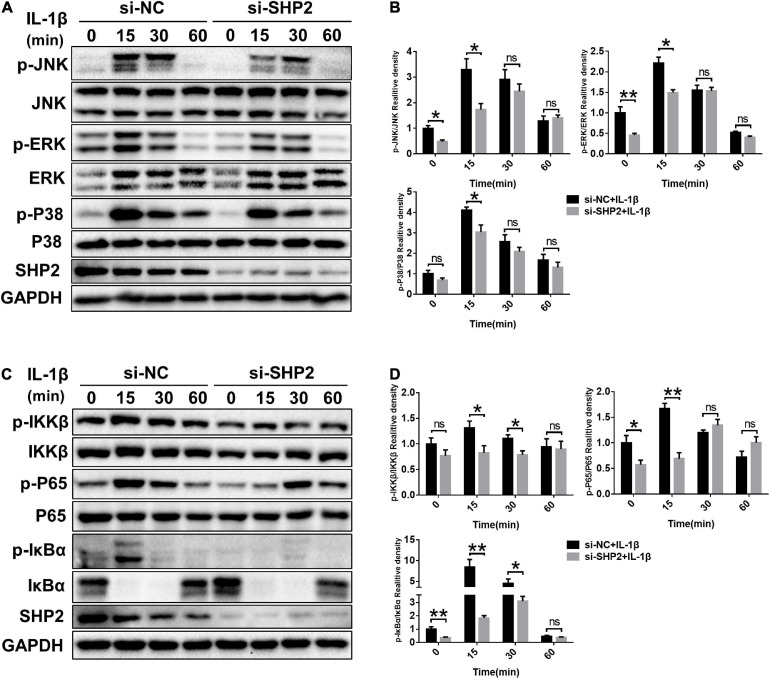
SHP2 knockdown inhibits MAPK and NF-κB signaling pathways. Chondrocytes were transfected with siRNA for 48 h, and then stimulated with IL-1β for indicated time points. **(A)** Representative western blot and **(B)** quantification of the effect of SHP2 silencing on phosphorylated p38, JNK, ERK in chondrocytes. **(C)** Representative western blot and **(D)** quantification of the effect of SHP2 silencing on phosphorylated p65, IKKβ, and IκBα in chondrocytes. *P < 0.05, **P < 0.01 versus cells transfected with si-NC and without IL-1β treatment.

**FIGURE 6:**
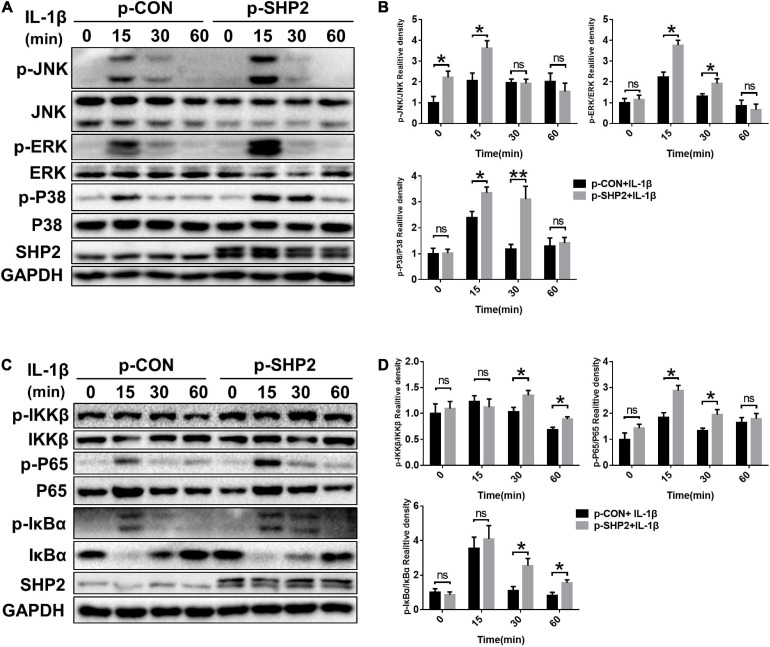
SHP2 overexpression activates MAPK and NF-κB Signaling Pathways. Chondrocytes were transfected with p-CON or p-SHP2 overexpression plasmid for 48 h, and then stimulated with IL-1β for indicated time points. **(A)** Representative western blot and **(B)** quantification of the effect of SHP2 overexpression on phosphorylated p38, JNK, ERK in chondrocytes. **(C)** Representative western blot and **(D)** quantification of the effect of SHP2 overexpression on phosphorylated p65, IKKβ, and IκBα in chondrocytes. *P < 0.05, **P < 0.01 versus cells transfected with p-CON and without IL-1β treatment.

The MAPK and NF-κB signaling pathways, widely involved in OA pathophysiology through various effects, are activated in osteoarthritic chondrocytes during aging and inflammation (Chang et al., 2019; Wongwichai et al., 2019). Hence, to investigate whether SHP2 influences IL-1β induced cartilage degradation via NF-κB and MAPK signaling pathways, we examined the phosphorylation levels of components of the MAPK and NF-κB pathways in chondrocytes. As shown in Figures 5A,B, the phosphorylated JNK, ERK, p38 protein levels were diminished in IL-1β stimulated chondrocytes following SHP2 siRNA transfection. The NF-κB pathway-related proteins were also tested. Phosphorylated IKKβ, p65 and IκBα were apparently decreased (Figures 5C,D). In contrast, overexpression of SHP2 significantly elevated the level of phosphorylated JNK, ERK, and p38 induced by IL-1β (Figures 6A,B). Phosphorylated IKKβ, p65, and IκBα were also increased after SHP2 overexpression (Figures 6C,D). These results implicated that SHP2 induced inflammation by activating MAPK and NF-κB signaling pathways.

### Knockdown of SHP2 Attenuates Cartilage Degradation in DMM Model

**FIGURE 7:**
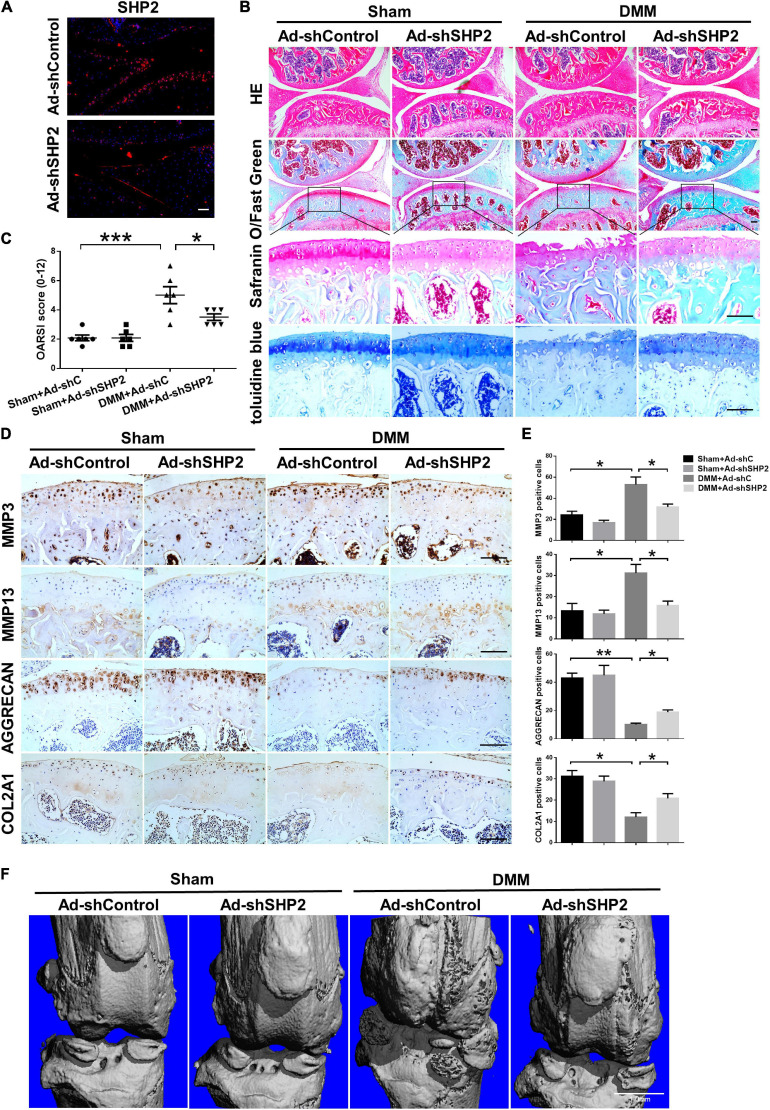
Knockdown of SHP2 prevents cartilage destruction at 8 weeks after DMM surgery. **(A)** Immunofluorescence image of SHP2 after mice were intra-articular injected with Ad-shControl and Ad-shSHP2 virus 8 weeks after DMM surgery, scale bar = 100 μm. **(B)** HE, Safranin O/Fast Green, and toluidine blue staining and **(C)** OARSI grades of knee joints from Ad-shControl and Ad-shSHP2 mice at 8 weeks after DMM surgery. Scale bars, 100 μm, *P < 0.05, ***P < 0.001 versus Sham + Ad-shC or DMM + Ad-shC group. **(D,E)** Immunostaining images and quantification of MMP3, MMP13, AGGRECAN, and COL2A1 in the joint cartilage of control and SHP2 knockdown mice at 8 weeks after DMM surgery. *P < 0.05, **P < 0.01 versus Sham + Ad-shC or DMM + Ad-shC group. *n* = 6. Scale bars, 100 μm. **(F)** Representative micro-CT images of the knee joints in each group. *n* = 6. Scale bar, 1.0 mm.

**FIGURE 8:**
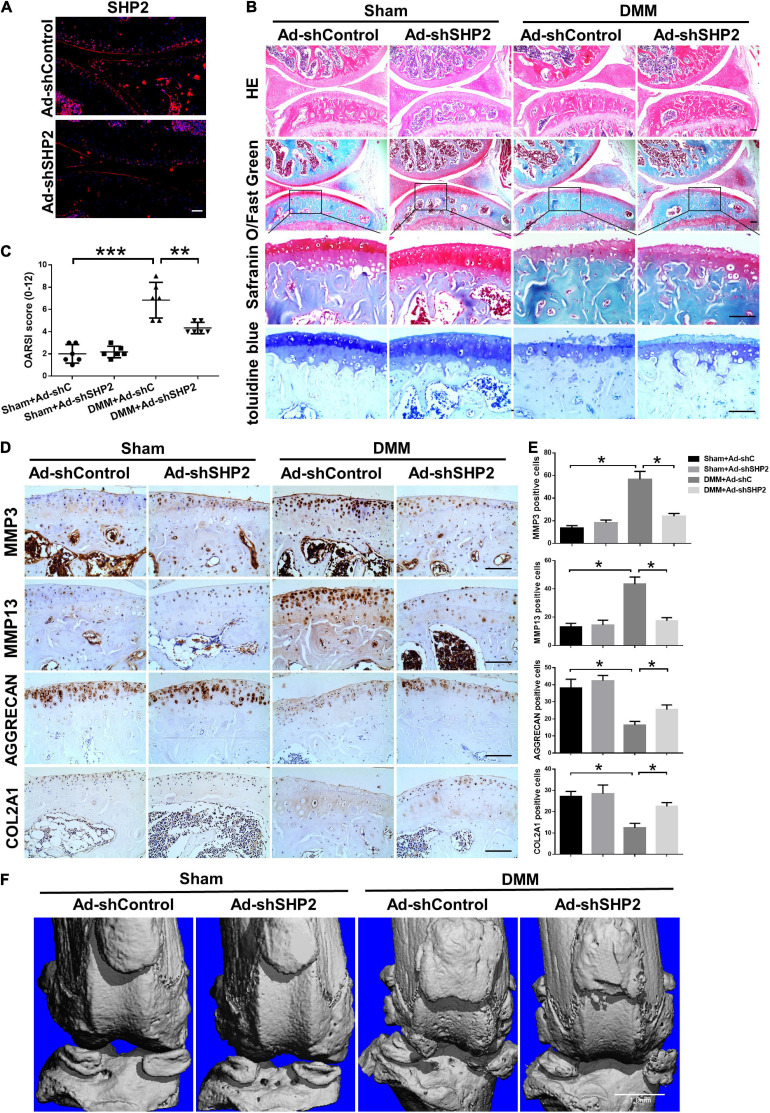
Knockdown of SHP2 prevents cartilage destruction at 12 weeks after DMM surgery. **(A)** Immunofluorescence image of SHP2 after mice were intra-articular injected with Ad-shControl and Ad-shSHP2 virus 12 weeks after DMM surgery, scale bar = 100 μm. **(B)** HE, Safranin O/Fast Green, and toluidine blue staining and **(C)** OARSI grades of knee joints from Ad-shControl and Ad-shSHP2 mice at 12 weeks after DMM surgery. Scale bars, 100 μm, **P < 0.01, ***P < 0.001 versus Sham + Ad-shC or DMM + Ad-shC group. **(D,E)** Immunostaining images and quantification of MMP3, MMP13, AGGRECAN, and COL2A1 in the joint cartilage of control and SHP2 knockdown mice at 12 weeks after DMM surgery. *P < 0.05 versus Sham + Ad-shC or DMM + Ad-shC group. *n* = 6. Scale bars, 100 μm. **(F)** Representative u-CT images of the knee joints in each group. *n* = 6. Scale bar, 1.0 mm.

To explore the possible association between SHP2 and OA *in vivo*, the mice OA model was induced by destabilized medial meniscus (DMM) surgery. A week after surgery, mice were intra-articular injected weekly with Ad-shControl or Ad-shSHP2 adenovirus both in sham-operated and DMM groups. Eight or twelve weeks after surgery, hematoxylin and eosin (HE), Safranin O/Fast Green, Toluidine blue staining and Immuno-histochemical staining were performed to assess the histomorphology differences among these groups. The efficiency of SHP2 knockdown in the cartilage was confirmed by SHP2 staining (Figure 7A). Data showed DMM group developed moderate cartilage destruction at 8 weeks after surgery. However, knockdown of SHP2 (Ad-shSHP2) dramatically alleviated cartilage degradation as demonstrated by Safranin O and Toluidine blue staining, with significantly lower OARSI grades at 8 weeks after DMM surgery (Figures 7B,C). In addition, the expression of MMP3 and MMP13 were significantly decreased in the DMM + shSHP2 group compared with the DMM + shControl group, while the levels of COL2A1 and AGGRECAN were significantly increased (Figures 7D,E). μCT was applied to view the osteophyte formation and determine the effects of SHP2 on the structure of tibial subchondral bone in these mice. More osteophytes were seen in DMM + Ad-shControl group, and SHP2 depletion reduced osteophyte formation at 8 weeks after surgery (Figure 7F). Also, we tested the effect of SHP2 in DMM model 12 weeks after surgery. Although osteophyte formation at 12 weeks seems to be less pronounced than that at 8 weeks after surgery in DMM + Ad-shControl group, SHP2 knockdown still ameliorated osteophyte formation and osteoarthritis progress (Figures 8A–F). The parameters of BV/TV, Tb.Th, and Tb.Sp were evaluated. Interestingly, SHP2 knockdown remarkably decreased BV/TV in sham-operated mice both at 8 and 12 weeks after surgery in subchondral bone, but no differences were seen in Tb.N, Tb.Th, and Tb.Sp. However, the difference in BV/TV was not observed between DMM + Ad-shSHP2 and DMM + Ad-shControl groups at 8 and 12 weeks after surgery (Supplementary Figure 2). The above results illustrated that inhibition of SHP2 reversed cartilage degradation and osteophyte formation *in vivo* without significantly affecting tibial subchondral bone remodeling in mice.

## Discussion

Degeneration of the articular cartilage is the main pathological feature of OA joints, which leads to cartilage damage, subchondral bone remodeling, and osteophyte formation (Loeser et al., 2012). To date, there is still no effective treatment for OA. In the past 20 years, SHP2 has been reported to participate in developmental diseases, such as Noonan syndrome, metachondromatosis, and malignancies such as juvenile myelomonocytic leukemia (Zhang et al., 2015). For example, two studies focused on the upregulation of SHP2 in cancer and concomitant enhancement in tumor invasion (Chauhan et al., 2000; Sturla et al., 2011). Therefore, we aim to identify the association between SHP2 and OA. In the present study, we found SHP2 expression was significantly induced by IL-1β treatment. By loss-of-function and gain-of-function studies in chondrocytes, we found that SHP2 apparently affected MMP3, MMP13, AGGRECAN, COL2A1, SOX9, and COL10A1 expression. These results strongly suggested SHP2 play an important role in cartilage degeneration. However, we found SHP2 had no significant effect on chondrocyte apoptosis, proliferation or migration, indicating the effect of SHP2 on cartilage degeneration was not through influencing chondrocyte function.

The canonical Wnt/β-catenin signal axis has been involved in the pathogenesis of OA (Corr, 2008). Researchers concluded that activation of the Wnt/β-catenin pathway resulted in articular chondrocytes degradation toward catabolism and loss of structure and function. Several studies demonstrated the relation between SHP2 and β-catenin. Ilse Timmerman et al. reported that SHP2 regulated the restoration of endothelial cell adhesion junctions by regulating β-catenin phosphorylation (Timmerman et al., 2012). However, how SHP2 regulate β-catenin was not studied in cartilage. Our data recognized a targeted β-catenin in OA progression. SHP2 knockdown reduced β-catenin level accompanied by decreased levels of target genes such as *Axin2*, *LEF1*, and *TCF4*. The interaction of SHP2 and β-catenin was identified by co-immunoprecipitation and double immune-staining. Besides, constitute activation of β-catenin reversed the decrease of catabolic enzymes MMP3 and MMP13 induced by knockdown of SHP2 after IL-1β stimulation in primary chondrocytes, showing that β-catenin was a direct target of SHP2 to induce chondral degeneration. Notably, as SHP2 is a protein tyrosine phosphatase, we found p-β-catenin (Y142) was not altered by SHP2. Therefore, we speculated Y142 site of β-catenin may not be the specific dephosphorylated site for SHP2 to interact with. Further investigations need to be performed to recognize the specific dephosphorylation site of SHP2 on β-catenin.

The inflammation status in cartilage or periarticular tissues is mostly attributed to MAPK and NF-κB pathways whose hyperactivation directly initiate and target articular cartilage and subchondral bone, causing cartilage degeneration and subchondral bone formation. Previous studies have shown SHP2 mediated MAPK and NF-κB activation (Zou et al., 2007; Heun et al., 2019; Niogret et al., 2019; Liu et al., 2020). However, whether SHP2 regulated these signaling in OA pathogenesis was not fully elucidated. Our data found that inhibition of SHP2 impaired MAPK and NF-κB pathways while overexpression of SHP2 significantly activated MAPK and NF-κB pathways after IL-1β exposure, indicated SHP2 indeed regulate inflammation status *in vitro*. This may be one of the mechanisms for a better understanding of the potential role of SHP2 in OA initiation and progression. Surprisingly, SHP2 knockdown or overexpression, have a significant impact on cartilage degeneration such as altered MMP3, MMP13 level and downstream MAPK, NF-κB pathways without IL-1β treatment (see IL-1β 0 h treatment). This may attribute to that SHP2 knockdown or overexpression already affected β-catenin signaling at baseline levels (Figures 3B,D). Altered β-catenin further affect downstream OA-like phenotype (Figure 2A) and MAPK, NF-κB pathways (Figures 5A–D). The similar phenomenon was not seen in our DMM models, where knockdown of SHP2 by intra-articular injection of Ad-shSHP2 robustly prevented cartilage degradation compared with the control both at 8 and 12 weeks after DMM surgery, but not in sham-operated mice. Since *in vivo* researches are much more complicated than *in vitro*, it is normal to see some of the inconsistency in different models. Accordingly, MMP3 and MMP13 levels decreased in Ad-shSHP2 injected mice confirmed its role in catabolic enzymes, the increased AGGRECAN and COL2A1 levels further proved its protective role in OA pathogenesis after SHP2 knockdown.

Interestingly, osteophyte formation was not so obvious as 8 weeks results, this maybe explained that the osteophyte formation rate was alleviated as time went by in all groups but SHP2 knockdown still has a beneficial effect on osteophyte formation at 12 weeks after surgery. Unlike cartilage degeneration process, most degenerated chondrocytes are hypertrophic differentiated and are kind of “irreversible” in OA pathogenesis. Osteophyte formation was a result of sclerosis of the subchondral bone, which should be faster to be alleviated. The current study found the subchondral bone mass was not significantly altered after SHP2 reduction as shown by μCT although SHP2 knockdown remarkably decreased BV/TV in sham-operated mice both at 8 and 12 weeks after surgery in subchondral bone, no differences were seen in Tb.N, Tb.Th, and Tb.Sp. Collectively, our results indicated that increased SHP2 is important for the initiation and progression of OA, SHP2 knockdown ameliorated cartilage degeneration.

Consistent with *in vitro* studies, there were some limitations of this study. First, the effect of overexpressing SHP2 *in vivo* was not detected although the *in vitro* results were shown in the present study, future researches will be conducted to see these results. Second, it would be more convincing if chondrocytes-specific SHP2 deletion mice can be used. However, since the construction and breeding of these mice are very time-consuming, we will extend this to animal models in the future.

Given the facts above, our study identified that SHP2 played a vital role in the degeneration of articular cartilage in OA. SHP2 knockdown protected cartilage from degradation during OA pathogenesis via suppressing the Wnt/β-catenin pathway, MAPK and NF-κB signaling pathways. These proved SHP2 might be a promising therapeutic target for OA.

## Data Availability Statement

The original contributions presented in the study are included in the article/Supplementary Material, further inquiries can be directed to the corresponding author/s.

## Ethics Statement

The animal study was reviewed and approved by the Ethics Committee on Animal Experimentation of Tongji Hospital, Huazhong University of Science and Technology. Written informed consent was obtained from the owners for the participation of their animals in this study.

## Author Contributions

JX and XS designed the study. TT performed most of the experiments. DL, CG, HL, and ZL helped with the DMM surgery. WL, CZ, and DQ helped inject the mice. ZD analyzed some of the data. All authors provided editorial comments.

## Conflict of Interest

The authors declare that the research was conducted in the absence of any commercial or financial relationships that could be construed as a potential conflict of interest.

## Supplementary Material

Supplementary Figure 1 The impact of SHP2 on cell migration, proliferation and apoptosis. Chondrocytes were seeded onto each well after transfection for 48 h, and then chondrocytes were incubated with or without IL-1β (5 ng/mL) for 24 h. **(A,B)** An apoptosis detection kit was used to detect chondrocytes apoptosis by flow cytometry after si-NC or si-SHP2 transfection. Data are means ± SEM of three independent experiments; ^∗^*P* < 0.05 versus the control group. **(C,D)** Chondrocytes apoptosis by flow cytometry after p-Con or p-SHP2 transfection. Data are means ± SEM of three independent experiments; ^∗^*P* < 0.05 versus the control group. **(E,F)** The proliferation of chondrocytes was detected by CCK-8 assay. **(G–J)** The scratch-wound assay and Quantitative data revealed the impacts of transfection with SHP2 siRNA or overexpression plasmid on the migration of chondrocytes. Scale bar, 100 μm. Data are means ± SEM of three independent experiments; ^∗^*P* < 0.05 versus the control group.

Supplementary Figure 2 The effect of SHP2 on subchondral bone quality in mice. **(A)** μCT parameters of BV/TV, Tb. N, Tb. Th and Tb. Sp of subchondral bone in each group of mice 8 weeks after DMM surgery. *n* = 6, ^∗^*P* < 0.05 versus Sham+Ad−shControl group. **(B)** uCT parameters of BV/TV, Tb. N, Tb. Th and Tb. Sp of subchondral bone in each group of mice 12 weeks after DMM surgery. *n* = 6, ^∗^*P* < 0.05 versus Sham+Ad−shControl group.
