# Supplementary figures and images for: Src Homology 2 Domain-Containing Protein Tyrosine Phosphatase Promotes Inflammation and Accelerates Osteoarthritis by Activating β-Catenin
Source: Front Cell Dev Biol. 2021 Apr 9;9:646386. doi: 10.3389/fcell.2021.646386 (PMC8063055; doi:10.3389/fcell.2021.646386)

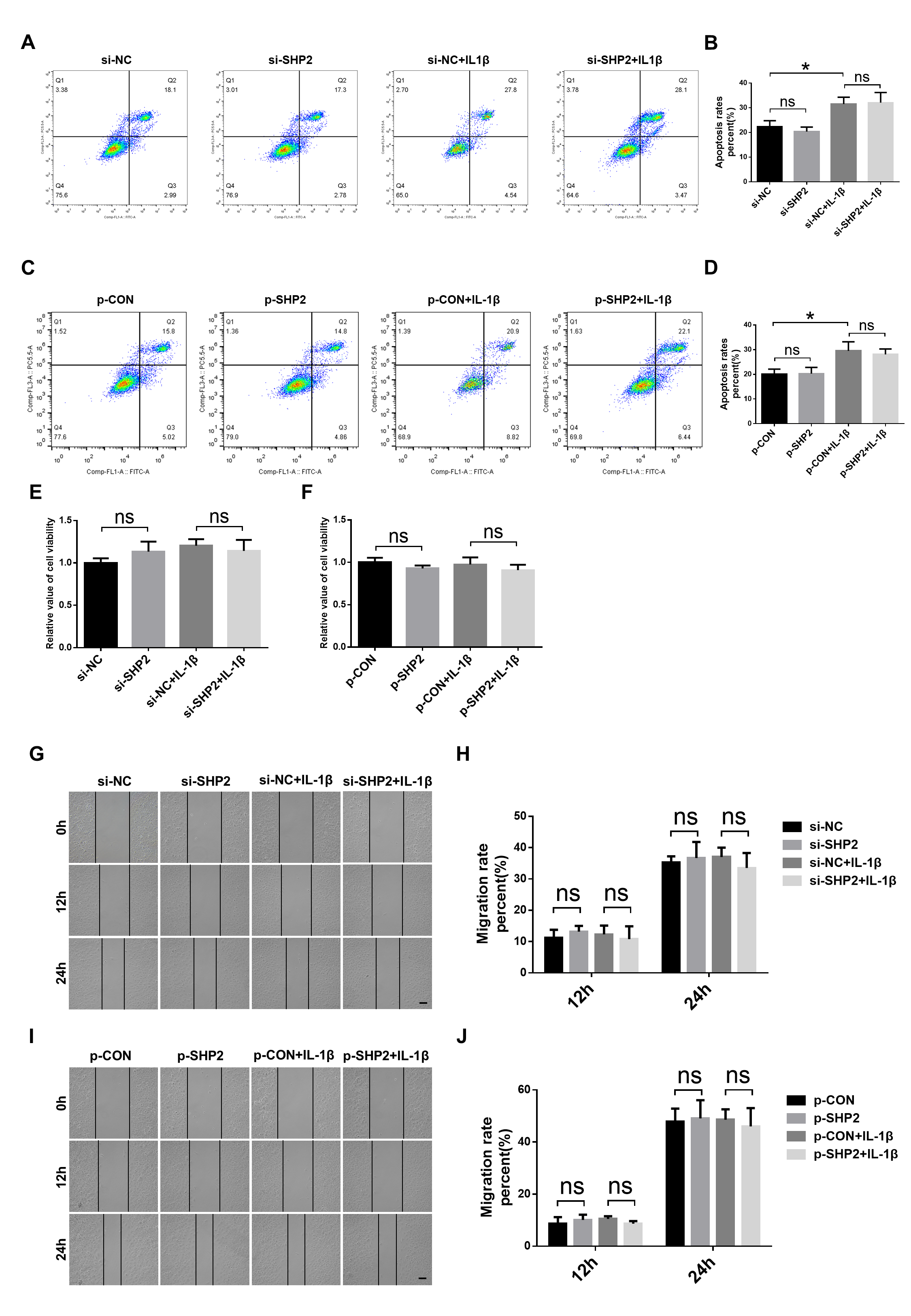

Supplement: Supplementary Figure 1 — The impact of SHP2 on cell migration, proliferation and apoptosis. Chondrocytes were seeded onto each well after transfection for 48 h, and then chondrocytes were incubated with or without IL-1β (5 ng/mL) for 24 h. (A,B) An apoptosis detection kit was used to detect chondrocytes apoptosis by flow cytometry after si-NC or si-SHP2 transfection. Data are means ± SEM of three independent experiments; ∗P < 0.05 versus the control group. (C,D) Chondrocytes apoptosis by flow cytometry after p-Con or p-SHP2 transfection. Data are means ± SEM of three independent experiments; ∗P < 0.05 versus the control group. (E,F) The proliferation of chondrocytes was detected by CCK-8 assay. (G–J) The scratch-wound assay and Quantitative data revealed the impacts of transfection with SHP2 siRNA or overexpression plasmid on the migration of chondrocytes. Scale bar, 100 μm. Data are means ± SEM of three independent experiments; ∗P < 0.05 versus the control group. [file Image_1.TIF]

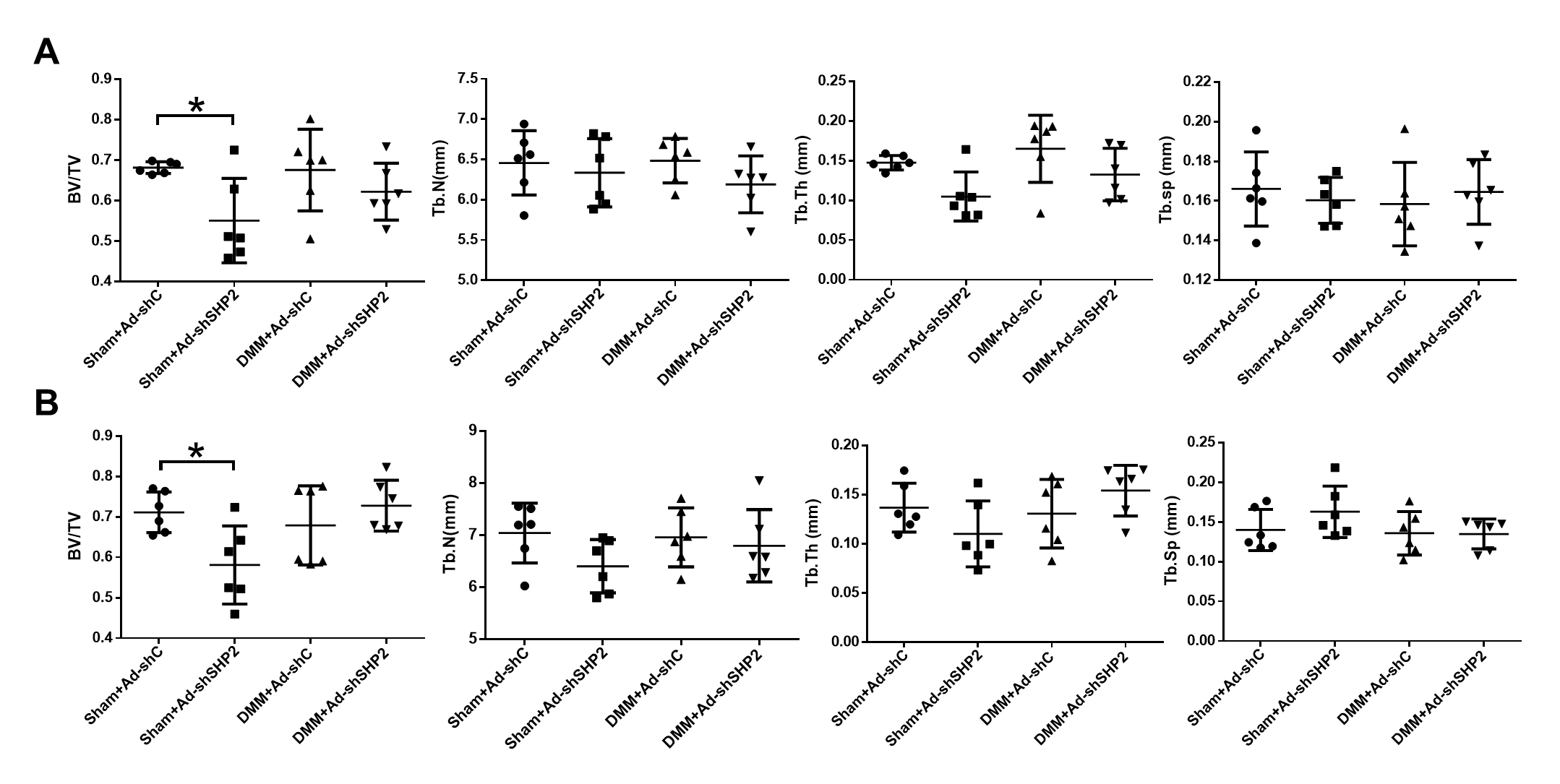

Supplement: Supplementary Figure 2 — The effect of SHP2 on subchondral bone quality in mice. (A) μCT parameters of BV/TV, Tb. N, Tb. Th and Tb. Sp of subchondral bone in each group of mice 8 weeks after DMM surgery. n = 6, ∗P < 0.05 versus Sham+Ad−shControl group. (B) uCT parameters of BV/TV, Tb. N, Tb. Th and Tb. Sp of subchondral bone in each group of mice 12 weeks after DMM surgery. n = 6, ∗P < 0.05 versus Sham+Ad−shControl group. [file Image_2.tif]
